# X-Ray Crystal Structure of the Full Length Human Chitotriosidase (CHIT1) Reveals Features of Its Chitin Binding Domain

**DOI:** 10.1371/journal.pone.0154190

**Published:** 2016-04-25

**Authors:** Firas Fadel, Yuguang Zhao, Alexandra Cousido-Siah, Francesc X. Ruiz, André Mitschler, Alberto Podjarny

**Affiliations:** 1 Department of Integrative Biology, Institute of Genetics and Molecular and Cellular Biology (IGBMC), CNRS UMR 7104, INSERM U 964, Université de Strasbourg, Illkirch, France; 2 Division of Structural Biology, University of Oxford, The Henry Wellcome Building for Genomic Medicine, Headington, Oxford, United Kingdom; University of Graz, AUSTRIA

## Abstract

Chitinases are enzymes that catalyze the hydrolysis of chitin. Human chitotriosidase (CHIT1) is one of the two active human chitinases, involved in the innate immune response and highly expressed in a variety of diseases. CHIT1 is composed of a catalytic domain linked by a hinge to its chitin binding domain (ChBD). This latter domain belongs to the carbohydrate-binding module family 14 (CBM14 family) and facilitates binding to chitin. So far, the available crystal structures of the human chitinase CHIT1 and the Acidic Mammalian Chitinase (AMCase) comprise only their catalytic domain. Here, we report a crystallization strategy combining cross-seeding and micro-seeding cycles which allowed us to obtain the first crystal structure of the full length CHIT1 (CHIT1-FL) at 1.95 Å resolution. The CHIT1 chitin binding domain (ChBD_CHIT1_) structure shows a distorted β-sandwich 3D fold, typical of CBM14 family members. Accordingly, ChBD_CHIT1_ presents six conserved cysteine residues forming three disulfide bridges and several exposed aromatic residues that probably are involved in chitin binding, including the highly conserved Trp465 in a surface- exposed conformation. Furthermore, ChBD_CHIT1_ presents a positively charged surface which may be involved in electrostatic interactions. Our data highlight the strong structural conservation of CBM14 family members and uncover the structural similarity between the human ChBD_CHIT1_, tachycitin and house mite dust allergens. Overall, our new CHIT1-FL structure, determined with an adapted crystallization approach, is one of the few complete bi-modular chitinase structures available and reveals the structural features of a human CBM14 domain.

## 1. Introduction

Carbohydrate-protein interactions and carbohydrate catalysis have attracted significant attention due to their importance in numerous biological processes, such as cell-cell recognition and cell adhesion among others. Protein domains involved in such interactions are non-catalytic modules called carbohydrate-binding modules (CBMs) that can be associated to carbohydrate-degrading enzymes [1–2] and are thought to promote binding of insoluble carbohydrate polymers, thus increasing the efficiency of the catalytic activity [3–11]. Currently, there are 64 families of CBMs classified according to amino acid sequence similarity in the CAZy database [12–13] but which can be structurally diverse [14].

Chitin consists of a linear β-1,4-linked polymer of N-acetylglucosamine (GlcNAc) and is highly insoluble. It is the second most abundant natural polysaccharide after cellulose and is a major component of fungal cell walls, including those of plant and human pathogens [15]. Chitinases [EC 3.2.1.14] are glycoside hydrolases (GH) that catalyze the hydrolytic degradation of chitin. They are classified in two families, GH18 and GH19, which differ in structure and mechanism. GH18 chitinases have been identified in a large number of organisms varying from lower organisms to humans. A large number of GH18 chitinases are multi-modular meaning that, in addition to their catalytic domains, they contain one or more additional domains. Among these, we find the chitin binding domains (ChBDs), belonging to different CBM families and enabling a more efficient substrate hydrolysis [16–18]. ChBDs are most commonly located at the C-terminus of the catalytic domain linked by a hinge region. Of the ChBDs that are associated with a chitinase catalytic domain, CBM5 and CBM12 are the most extensively studied. They are usually found in bacteria. CBM18 almost exclusively includes ChBDs from plants, with the exception of one CBM18 identified in *Streptomyces griseus* [19]. On the other hand, CBM14 is commonly present in chitinases from *baculoviridae*, invertebrates, and mammals including humans. CBM14 and CBM18 can be expressed solely as an individual module or linked to a chitinase catalytic domain.

Chitotriosidase-1 (CHIT1) and acidic mammalian chitinase (AMCase) are the only active human chitinases. They are composed of a GH18 catalytic domain linked by a hinge to a CBM14 ChBD. The crystal structures of their catalytic domains have been determined [20–22]. CHIT1 is involved in the innate immune response against chitin-containing pathogens [23] and is also produced by macrophages and neutrophils [24–26]. CHIT1 can exist in two isoforms, a 39 kDa lysosomal isoform (with the catalytic domain only) and the full-length 50 kDa secreted isoform (CHIT1-FL). This last isoform has been detected in Gaucher disease, where its expression increases between 10–1000 fold. Additionally, CHIT1 is upregulated in patients suffering from infections, chronic inflammation or degenerative disorders [27–28]. Although CHIT1 has been well-characterized as a clinical marker, its specific function and effects under normal and pathological conditions remain not fully understood. Interestingly, recent studies have inferred the interaction of CHIT1 with glycan substrates associated to the surface of epithelial cells and macrophages [29], and have implicated CHIT1 ChBD (ChBD_CHIT1_) in tumor metastasis of osteolytic lesions [30]. Thus, the determination of the structural characteristics of the CHIT1-FL is essential to get new insights of its mode of action. Here, we report the crystal structure of CHIT1-FL at 1.95 Å resolution, including its CBM14 domain, determined with an adapted crystallization approach combining cross-seeding and micro-seeding screening cycles. Our structure is one of the few complete bi-modular chitinase structures available in addition to ChiA and ChiB from *Serratia marcescens* [31–32]. Our structural and evolutionary analysis reveals a high mobility of ChBD_CHIT1_, mediated by the flexible linker region, and highlights the importance of the conserved residues in maintaining the functionality of ChBD_CHIT1_.

## 2. Materials and Methods

### 2.1. Cloning, expression and purification

Human CHIT1-FL cDNA (GenBank: BC105682) of the 50 kDa CHIT1 isoform was used as a template to generate the C-terminal thrombin site and His-tag by two polymerase chain reactions (PCR) using the following primers (SIGMA): 5’-AATTCAAGCTTGCCACCATGGTGCGGTCTGTGG-3’ (N-terminal derived sense primer) and two antisense primers 5’-GTGATGGTGATGGTGGTGAGAACCGCGTGGCACCAGATTCCAGGTGCAGCATTTG-3’; 5’-ATTATCGCGATACTAGTCTCGAGTCATTAGTGATGGTGATGGTGGTG-3’. The final PCR product was cloned into the pHL expression vector [33]. CHIT1-FL was transiently expressed in adherent HEK293T cells grown in roller bottles in the presence of the N-glycosylation inhibitor kifunensine [34] as previously described [33]. After dialysis against 25 mM phosphate buffer saline (PBS) pH 8.0 at 4°C, the secreted protein was purified from the media using an immobilized metal affinity chromatography (IMAC) batch procedure. CHIT1-FL was further purified by size exclusion chromatography on a Superdex 200 HR 16/60 (GE Healthcare) in 10 mM HEPES, 150 mM NaCl pH 7.5. The protein purity was assessed by SDS–PAGE (0.1% SDS, 12.5% polyacrylamide) [35] followed by Coomassie Brilliant Blue staining. The enzyme concentration was determined from the absorption at 280 nm using an UV NanoDrop 1000 Spectrophotometer (Thermo Scientific). The molar extinction coefficient was calculated using the ProtParam tool on the ExPasy server [36] to be 83935 M^-1^ cm^-1^.

### 2.2. Crystallization, cross-seeding and micro-seeding

A Tecan Temo 96 head robot (Tecan) was used to set up sparse matrix screen containing commercially available crystallization reagents such as the PEGS suite (Qiagen), Classics Lite suites (Qiagen), Index (Hampton Research) and MPD (Qiagen). The initial crystallization trials of CHIT1-FL were performed using a Mosquito crystallization robot (TTP LabTech) to set up sitting drops composed of 0.1 μl protein solution mixed with an equal volume of reservoir solution equilibrated against 40 μL of the reservoir solution. Although hundreds of crystallization conditions were tested, none of them succeeded. Next, we tried cross-seeding using micro-crystals of the CHIT1 39 kDa catalytic domain (CHIT1-CAT) as previously described by Fadel et al [21]. CHIT1-CAT crystals were crushed and used for automated high throughput cross-seeding screens. Each sitting drop consisted of 0.1 μl of the screening reservoir solution with 0.07 μl of the CHIT1-FL solution at 9 mg…ml^-1^ and 0.03 μl of the seeding stock. The drops were equilibrated against 40 μL of reservoir screen solution at 20°C. The first CHIT1-FL crystals appeared in the crystallization condition A (15% Polyethylene glycol (PEG) 3350, 0.2 M sodium sulfate) and had a highly anisotropic X-ray diffraction pattern. Crystals grown in condition A were used as seeds for a new Microseed Matrix Screening (MMS) with the Silver Bullets screen (Hampton Research), using in the reservoir 25% PEG 3350, 0.02 M HEPES pH 6.8. New CHIT1-FL crystals obtained in the F6 condition of the Silver Bullets screen showed good diffraction quality and were optimized manually by hanging drop vapor diffusion experiments. The final improved F6 condition consisted in drops composed of 1 μL of the F6 condition of the Silver Bullets additive containing 0.2% w/v 2-Methyl-2,4-pentanediol; 0.2% w/v 1,2,3-Heptanetriol; 0.2% w/v Diethylenetriaminepentakis (methylphosphonic acid); 0.2% w/v D-Sorbitol; 0.2% w/v Glycerol; Buffer 0.02 M HEPES pH 6.8 were added to 2 μl of the reservoir solution B (15% PEG 3350, 0.02 M HEPES pH 6.8), with 1 μL of the CHIT1-FL solution (8 mg ·ml^-1^ in 0.01 M HEPES pH 7.5, 0.15 M NaCl) and 0.5 μL of the micro-seeding stock prepared from the last screening round. The drops were equilibrated at 17°C against 500 μL of the reservoir containing solution B.

### 2.3. Cryo-cooling, data collection and molecular replacement

Crystals of CHIT1-FL grown in condition A were cryo-protected by sequential incubation for 30 seconds in two solutions containing increasing concentration of ethylene glycol (15% and 25%) in 20% PEG 3350, 0.2 M sodium sulfate pH 7.2, prior to flash-cooling in liquid nitrogen. Analogously, crystals of CHIT1-FL grown in optimized condition F6 were cryo-protected by sequential incubation for 30 seconds in two solutions containing increasing concentration of glycerol (15% and 25%) in 15% PEG 3350, 0.02 M HEPES pH 6.8, prior to flash-cooling in liquid nitrogen.

Data sets were collected at the Swiss Light Source (SLS) synchrotron on the X06DA (PXIII) beamline. After the optimization of the procedure, 800 diffraction images were collected using a Pilatus 2M detector, up to a resolution of 1.95 Å, with an oscillation range of 0.25° and an exposure time of 0.3 s per frame, with a none attenuated beam of a 1.0 Å X-ray wavelength. All data sets were integrated, merged and scaled using the programs HKL-2000 [37] and XDS [38]. The structure was solved by molecular replacement (MR) with Phaser [39] using the coordinates of the catalytic domain of the same protein as an initial search model (Protein Data Bank (PDB) ID 4WJX [21]). The model was improved by alternating cycles of manual model building using Coot [40] and refined using REFMAC5 [41] and PHENIX [42]. The stereochemical quality of the final model was assessed with MolProbity [43]. Structural figures were prepared using PyMOL (http://www.pymol.org). A summary of the data-collection processing and structure-refinement statistics is given in Table 1.

**Table 1:** Data collection and refinement statistics.

	CHIT1-FL
**PDB code**	5HBF
**Synchrotron, beamline**	SLS, X06DA (PXIII)
**Wavelength (Å)**	1.0
**Resolution range (Å)**	44.69–1.95 (2.01–1.95)
**Space group**	P 1 2_1_ 1
**Unit cell (Å,°)**	a = 51.14 b = 106.66 c = 85.67α = γ = 90 β = 107.13
**Total reflections**	242490 (22495)
**Unique reflections**	62392 (5738)
**Multiplicity**	3.9 (3.9)
**Completeness (%)**	96.63 (89.40)
**Mean I/sigma(I)**	15.06 (1.85)
**Wilson B-factor (Å**^**2**^**)**	29.66
**R-sym**	0.057 (0.63)
**R-meas**	0.077 (0.853)
**CC(1/2)**	0.998 (0.686)
**R-factor**	0.2051 (0.3605)
**R-free**	0.2454 (0.4082)
**Total number of atoms**	6951
**macromolecules**	6750
**Water**	177
**Protein residues**	849
**RMS (bonds, Å)**	0.007
**RMS (angles,°)**	1.10
**Ramachandran favored (%)**	97
**Ramachandran outliers (%)**	0
**Clashscore**	7.79
**Average B-factor (Å**^**2**^**)**	31.60
**macromolecules**	31.60
**solvent**	29.60

Statistics for the highest-resolution shell are shown in parentheses.

### 2.4. Electrospray Ionization Mass Spectrometry

Prior to Electrospray Ionization Mass Spectrometry (ESI-MS) analysis, CHIT1-FL was desalted on Zeba Spin Desalting Columns (Pierce) in 50 mM ammonium acetate. ESI-MS measurements were performed on an electrospray time-of-flight mass spectrometer (MicroTOF, BrukerDaltonic). Purity of the protein was verified by mass spectrometry (MS) in denaturing conditions (samples were diluted at 2 pmol μL^-1^ in a 1:1 water–acetonitrile mixture (v/v) acidified with 1% formic acid).

### 2.5. Structural Conservation Analysis

Homologous sequences to ChBD_CHIT1_ were obtained by BLAST (or PSI-BLAST) with an inclusion threshold of e  =  0.0001 in UniRef90 [44–45]. Alignments of sequences were performed using MAFFT [46]. The amino acid sequences used are given in the supplementary data. The rate of evolution at each site is calculated using the empirical Bayesian [47]. Structural conservation analysis was performed using the ConSurf server [48–49].

## 3. Results and Discussion

### 3.1. Crystallization of CHIT1-Full Length (CHIT1-FL)

**Fig 1:**
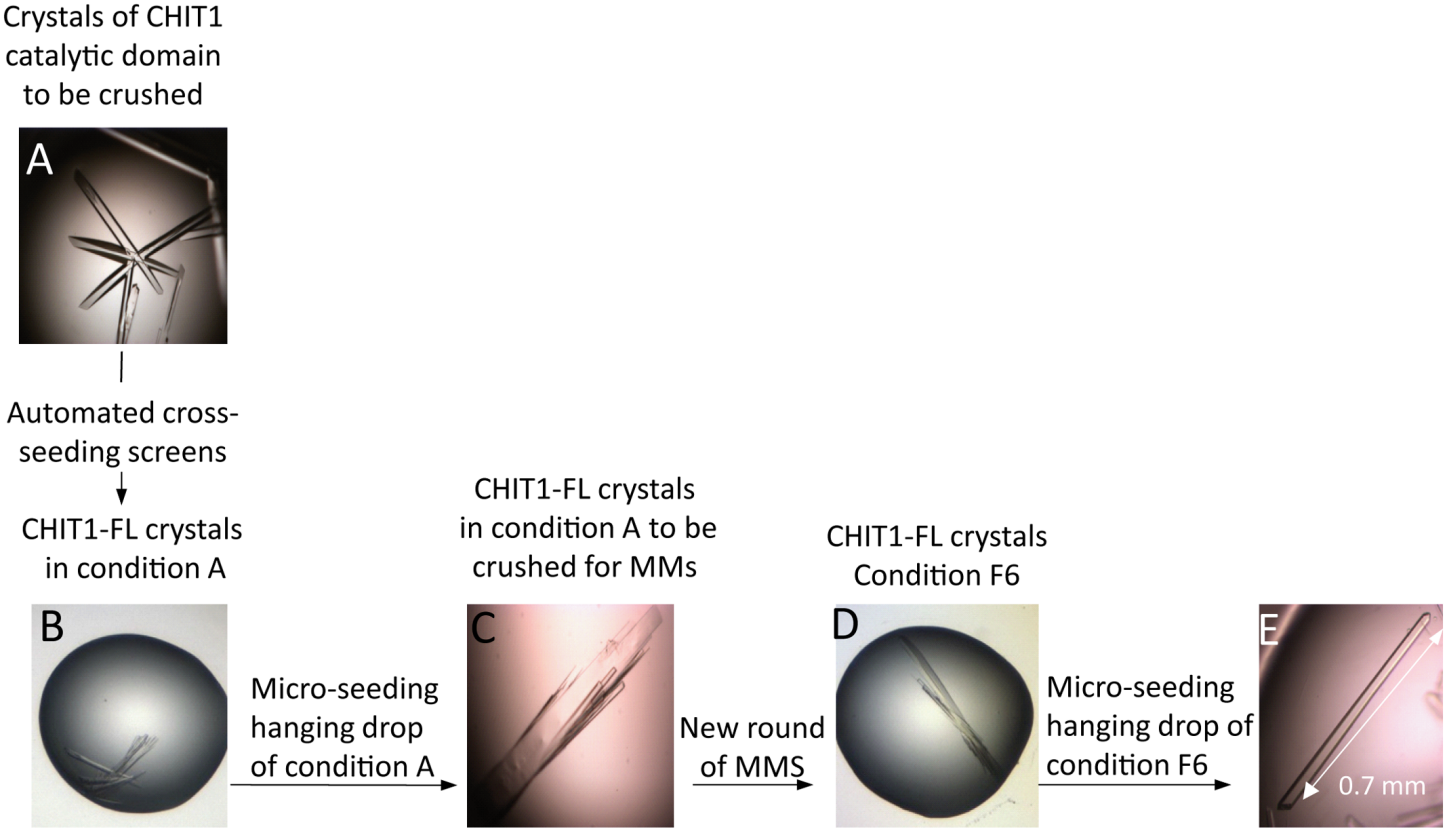
CHIT1-FL crystallogenesis strategy. (A) CHIT1-CAT crystals crushed and used for initial automated cross-seeding. (B) CHIT1-FL crystals obtained after the first cross-seeding round in crystallization condition A. (C) CHIT1-FL crystals obtained after optimization through manual hanging drop. (D) CHIT1-FL crystals from condition A crushed and used for another cycle of automated micro-seeding leading to crystallization condition F6. (E) CHIT1-FL crystals obtained after optimizing F6 condition.

With the aim to determine the structure of the CHIT1-FL enzyme and after testing a large number of unsuccessful crystallization conditions, we found a strategy that promoted crystallization of this enzyme: cross-seeding crystals from the CHIT1-CAT construct to induce the crystallization of the full length protein (Fig 1). The first crystals of CHIT1-FL construct were obtained in crystallization condition A. Despite their highly anisotropic diffraction pattern, datasets from these crystals were obtained at 2.6 Å resolution, in space group P2_1_2_1_2_1_,with unit-cell parameters a = 85.95, b = 108.30, c = 106.05 Å. The structure solved by MR showed two protein molecules in the asymmetric unit (AU) but displayed electron density corresponding only to the catalytic domain without any clear electron density for the hinge and the ChBD, posing the intrinsic difficulty of solving the X-ray structure of such a multi-domain protein. This prompted us to launch a new cycle of MMS using crystals from crystallization condition A. As detailed in Materials and Methods, we obtained new crystals in the condition F6 of the Silver Bullets screen (Hampton Research), which resulted in good diffraction quality in terms of decreasing anisotropy and mosaicity. Data from the best crystal obtained in this condition were processed at 1.95 Å resolution in a new space group P2_1_ with unit-cell parameters a = 51.12, b = 106.66, c = 85.66 Å, α = γ = 90, β = 107.11°. SDS-PAGE analysis of the dissolved CHIT1-FL crystals confirms that no proteolysis occurred in the drop (S1A Fig). The size-exclusion chromatography and native MS data confirm that the CHIT1-FL is monomeric in solution (S1B and S1C Fig). This means that the observed CHIT1-FL dimer was due to crystal packing contacts of the space group P2_1_, which resulted in the stabilization of the ChBD_CHIT1_.

Our results demonstrate that CHIT1-FL crystallization was a challenging task due to the flexibility of the hinge region linking the catalytic domain to the ChBD (discussed in the next paragraph). Remarkably, the crystals obtained by cross-seeding were able to induce the growth of crystals with a different space group of lower symmetry, thereby improving the packing and the diffraction pattern. The current case reinforces the notion that an exact match of crystal unit cells is not required for effective nucleation, as previously discussed by Shaw Stewart et al. [50]. The application of several cycles of micro-seeding experiments combined with automated high-throughput crystallization, with each cycle improving the quality of the crystals, allowed us to solve the so far elusive CHIT1-FL structure at 1.95 Å resolution.

### 3.2. Analysis of the crystal contacts and packing

**Fig 2:**
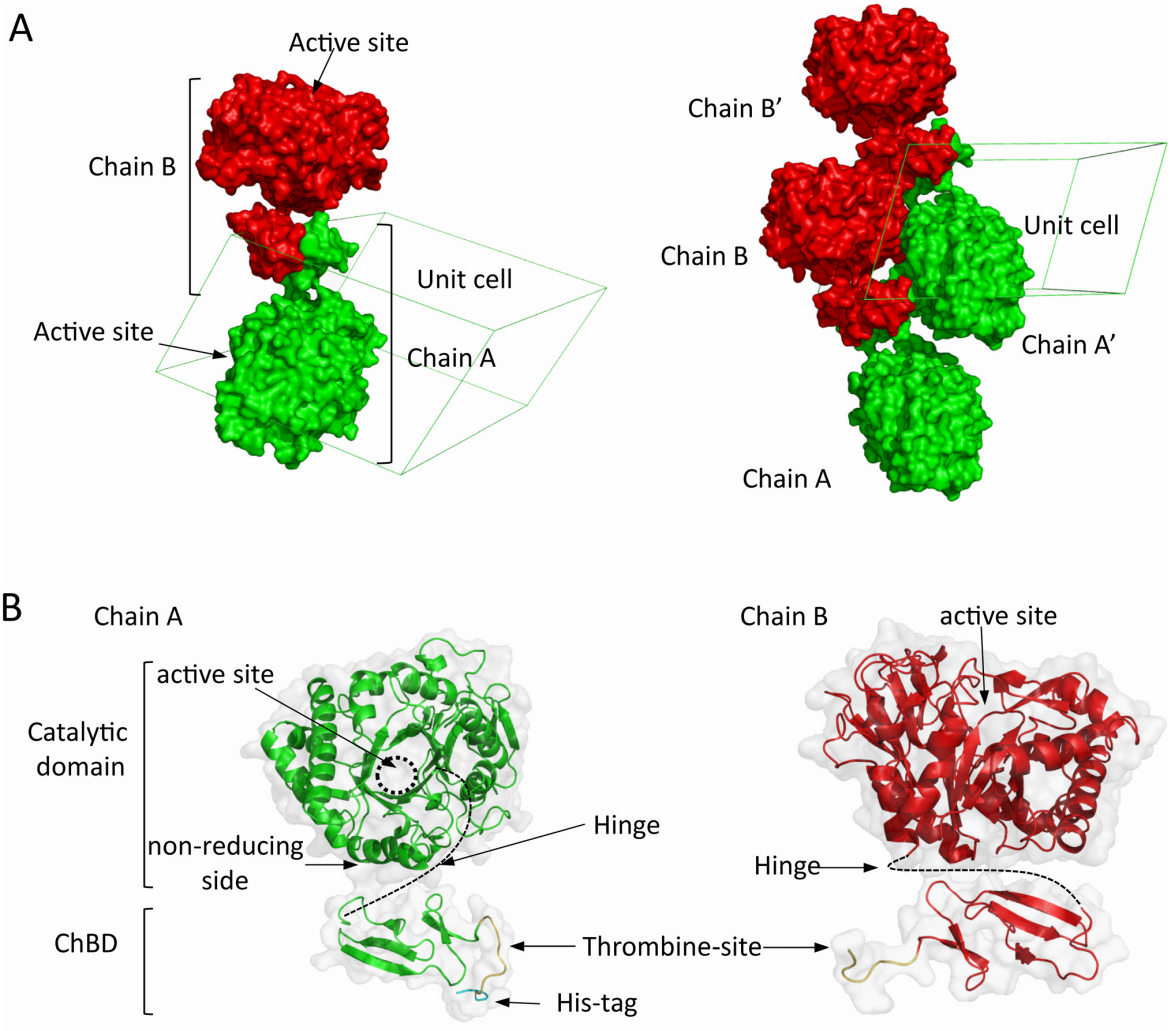
3D structure of CHIT1-FL. (A) Left, surface representation of two CHIT1-FL molecules in the unit cell (chain A and B). The position of the active site of each monomer show that they are not in the same direction. Right, surface representation of 4 CHIT1-FL monomers crystal packing. (B) Surface and ribbon representation of chain A and chain B comprising the catalytic and ChBD domains. Thrombin-site (yellow), His-tag (light violet) and hinge region (dotted line).

The X-ray structure of CHIT1-FL was solved by MR using the CHIT1-CAT (PDB ID 4WJX) as a search model. Data collection and refinement statistics are presented in Table 1. The final refined structure has R_work_ and R_free_ values of 20.51 and 24.54% respectively. Four disconnected domains appear in the AU: two differently oriented catalytic domains (Ala22-Leu386) and two ChBDs (Asn417-Asn466), corresponding to two CHIT1-FL monomers (Fig 2A and 2B and S2 Fig).

The hinge region, consisting of 31 residues is a Proline-rich region (9 Proline residues among 31) linking the catalytic domain to the ChBD, could not be modeled due to a lack of interpretable electron density. We thus wondered how the two ChBDs in the AU (with RMSD value of 0.24 Å) are paired to diverging catalytic domains to form two CHIT1-FL monomers. First, we noticed that the non-cleaved thrombin-site showed a distinct conformation at the C-terminus of the ChBD in chains A and B. Second, the electron density corresponding to four histidines from the His-tag was observed only on chain A. These two differences allowed us to distinguish each ChBD in the AU (Fig 2B) and to infer that the two ChBDs correspond to CHIT1-CAT monomers within the AU, instead of symmetry related copies. Then, based on the locations of CHIT1-CAT domain C-terminal / ChBD domain N-terminal residues, we analyzed the different CHIT1-CAT—ChBD_CHIT1_ pairing possibilities in the AU by calculating the free energy change (ΔG) of the interdomain contact (ΔG^int^) with PISA (http://www.ebi.ac.uk/msd-srv/pisa/cgi-bin/piserver?qa=4lyz). The most energetically favorable configuration (ΔG^int^ = -12.2 kcal mol^-1^ with a buried surface of 1063.4 Å^2^, see S3 Fig) is shown in Fig 2A and 2B. Indeed, the second next possible CHIT1-FL domain configuration shows two pseudo anti-parallel monomers with 813.5 Å^2^ of buried surface with a ΔG^int^ value of -1.7 kcal mol^-1^, rendering it energetically unfavorable in comparison to the first one.

The observation of remarkably high B-factors for ChBD_CHIT1_ (from 26 Å^2^ to ~80 Å^2^) in comparison to the B-factors for CHIT1-CAT domain (~30 Å^2^, S4 Fig) reinforces the idea of the high ChBD mobility mediated by the flexible hinge. This is an important difference when compared to other crystal structures of full length bacterial chitinases such as ChiA and ChiB from *Serratia marcescens* (S4 Fig), which display the CBM situated in a clearly defined orientation relative to the catalytic domain. For instance, ChiB has a C-terminal ChBD similar to CHIT1, but its hinge region is not flexible (average B-factor of 24.8 Å^2^) [32]. The low flexibility of the hinge in ChiB causes that its ChBD is located towards the C-terminus [32, 51]. On the other hand, ChiA has a N-terminal CBM extending to the substrate binding cleft. In addition, the two enzymes degrade the chitin polymer from different ends: ChiA acts from the reducing end while ChiB does it from the non-reducing end [32], [51]. Interestingly, Small Angle X-ray Scattering (SAXS) experiments on the cellulase endoglucanase D (EngD) showed that the positions of the CBM relative to the catalytic domain are quite variable in solution with no dominating conformation [52]. Thus, one could suggest that, as in the case of EngD, ChBD_CHIT1_ is not aligned with the catalytic domain but rather moves randomly affecting in turn the orientation of the catalytic domain. This is consistent with Abott *et al*. suggestion that linker regions devoid of secondary structure confer a random positioning (i.e. conformational flexibility) of the CBM with respect to the catalytic domain, facilitating coordinated substrate binding well-suited for structurally complex glycan environments, such as plant cell wall or mammalian mucosa [53]. Taken together, our data lead us to hypothesize that ChBD_CHIT1_ behaves as a probe inspecting the environment for the presence of substrate in a possible step-wise mechanism at the basis of CHIT1-FL action. When the ChBD_CHIT1_ locates the presence of chitin in the environment, it binds to it and then guides the catalytic domain to the substrate location. Once bound to chitin, the ChBD_CHIT1_ disrupts its crystalline structure making it accessible to be hydrolyzed by CHIT1-CAT.

### 3.3. Overall structure of ChBD_CHIT1_

**Fig 3:**
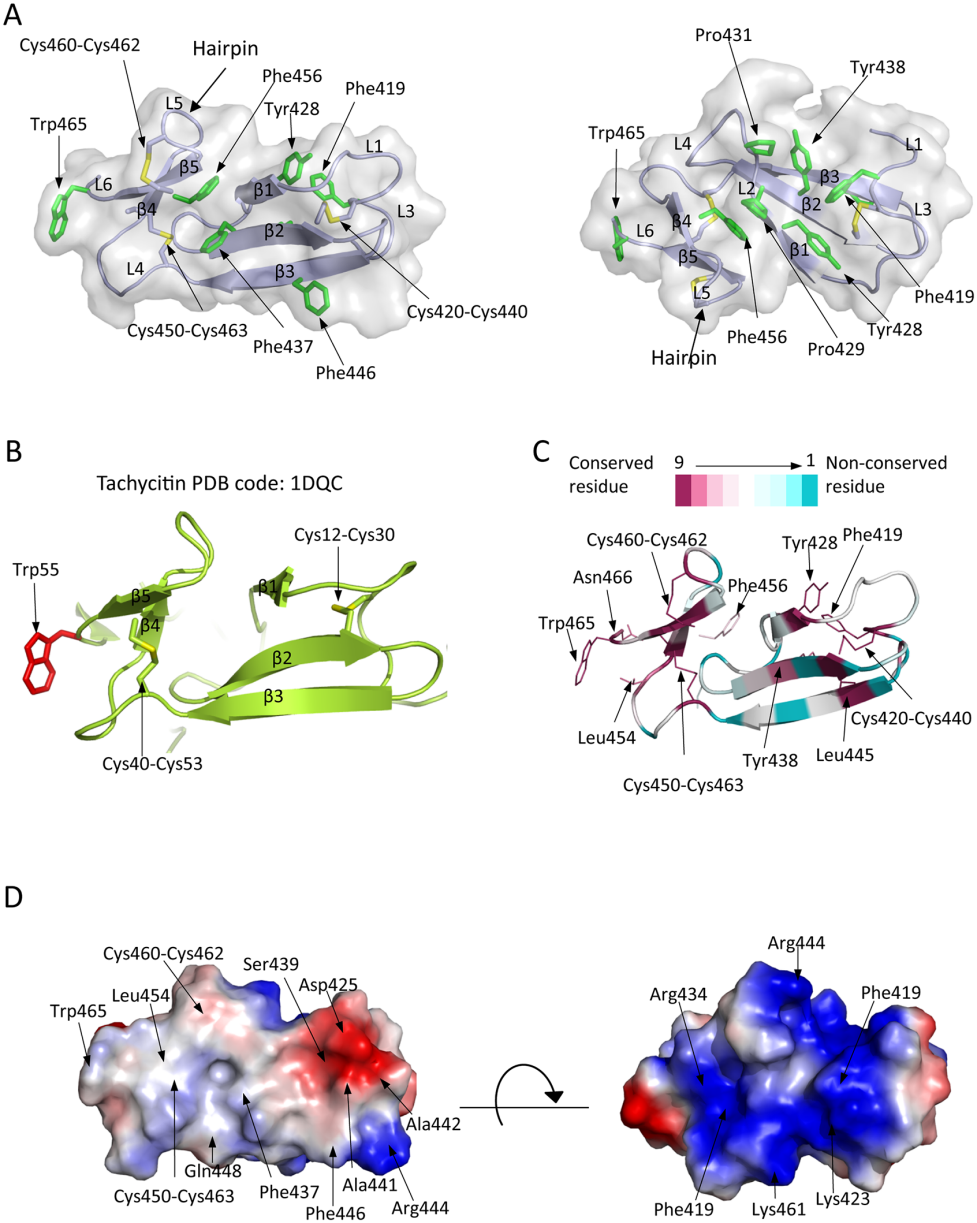
Structural and evolutionary features of ChBD_CHIT1_. (A) Solvent accessible surface of ChBD_CHIT1_ is shown in two orientations comprising its backbone represented as violet ribbons which illustrates the distorted β-sheet sandwich. The aromatic residues are labeled and represented as green sticks. The six cysteines forming disulfide bonds are represented as sticks where the sulfur atoms are colored in yellow. (B) Structural comparison with tachycitin. Ribbon representation of the tachycitin 3D structure with a distorted β-sheet sandwich fold. The conserved cysteins residues forming disulfide bonds are represented in sticks with the sulfur atom colored in yellow. Conserved Trp55 is shown as red stick. (C) Sequence conservation of ChBD_CHIT1_ is represented as ribbons and lines. Color-codes depend on the residue conservation degree (conserved, magenta to variable, cyan). Relevant conserved residues are represented in lines, labeled and indicated with arrows. (D) Representation of the electrostatic potential at the surface of ChBD_CHIT1_ in two orientations. The protein is shown as solvent-accessible surface colored by electrostatic potential at ± 5 kT/e. Color-codes depend on the electrostatic potential (red: negative charge; blue, positive charge; and white: neutral charge).

The crystal structure of ChBD_CHIT1_ comprises the last 49 C-terminal amino acids of the protein (417–466). In agreement with our ChBD_CHIT1_ structure, functional analysis defined those 49 residues as the minimal sequence required for chitin binding activity in CHIT1 [54]. The structure of the catalytic domain, which adopts the conserved (α/β)_8_ TIM barrel fold found in all GH18 family, is essentially the same as the already described CHIT1-CAT [20–21]. The structure of ChBD_CHIT1_ reveals an elongated conformation (dimensions 60 x 17 x 14 Å), which is different from the globular and compact conformation of ChBDs from bacteria and plants belonging to CBM5/12 and CBM18 respectively [4, 55]. The ChBD_CHIT1_ fold consists of a distorted β-sandwich composed of two β-sheets containing three N-terminal anti-parallel β-strands (β1, β2, β3; residues 427–428, 436–440, 445–449) and two C-terminal anti-parallel β-strands (β4, β5; 455–457, 460–464) (Fig 3A and 3B). By sequence similarity, ChBD_CHIT1_ has been attributed to the CBM14 family, which also exists in invertebrates e.g. insects and nematodes [56]. According to CAZy database[13], so far only three CBM14 structures from invertebrate organisms have been solved, two by NMR corresponding to tachycitin and the allergen Blo t 12 CBD (PDB IDs: 1DQC (Fig 3B) [57] and 2MFK), while the structure of allergen Der p 23 was solved by X-ray crystallography (PDB ID: 4ZCE) [58]. These three CBM14 exist as a single domain and are not linked to a chitinase enzyme. The sequence identity between ChBD_CHIT1_ with tachycitin, Blo t 12 and Der p 23 is 28%, 22.5% and 20.83% respectively. Structural comparison between the human ChBD_CHIT1_ and these three CBM14 structures reveals that they share the same distorted β-sandwich fold (Fig 3A, 3B and 3C), highlighting the conservation of the CBM14 folding from invertebrates to vertebrates.

Moreover, ChBD_CHIT1_ contains 6 cysteine residues [54] forming three disulfide bonds, as confirmed by our electron density map. The one between Cys420 and Cys440 connects the β-strand 2 (β2) to the beginning of the first loop (L1). The second disulfide bond is in the C-terminal region of ChBD_CHIT1_ (Cys450-Cys463) and links the last β5 with the L4 (Fig 3C). These two disulfide bonds exist in equivalent locations in tachycitin, Der p 23 and Blo t 12 CBD, suggesting that they are essential for the structural conservation of the overall CBM14 ChBD folding (Fig 3B and 3C). The remaining disulfide bond is established between two cysteine residues (Cys460-Cys462) linking and stabilizing the hairpin to the β5 (Fig 3A and 3C). Although these two latter cysteines do not exist in tachycitin, Der p 23 and Blo t 12 (solely ChBD), the evolutionary analysis performed by the Consurf server reveals that they are fully conserved in at least 150 ChBDs linked to chitinase catalytic domains and chitinase-like proteins in invertebrates and vertebrates (Fig 3C, S5 and S6 Figs). Interestingly, site-directed mutagenesis of these cysteine residues on CHIT1-FL have shown that each of them is critical for the binding activity to chitin [54]. This underlines the structural conserved role of the six cysteine residues to maintain the integrity of the ChBD in a functional folded conformation in chitinases containing the CBM14 domain.

### 3.4. Analysis of the ChBDCHIT1 aromatic residues and its electrostatic molecular potential surfaces

It is believed that the interaction of carbohydrate crystalline substrates, like chitin and cellulose, with their respective binding domains (ChBDs and cellulose binding domains (CBDs)) occurs via exposed aromatic residues [55, 59]. Although ChBD_CHIT1_ is a small module, it contains 7 aromatic residues among which 6 of them are exposed (Fig 3A and 3B). Indeed, within the core of the domain, there are 4 exposed aromatic residues: i) Phe437 and Phe446, located on β2 and β3 respectively and oriented to the same face of the domain (Fig 3A, left side); ii) Tyr428 and Tyr438, directed to the opposite face of ChBD_CHIT1_ (Fig 3A, right side). In this last region, Pro429 and Pro431 are facing each other, which could make this side an aromatic rich “canal-like” interface suitable for chitinous substrates binding. Moreover, the ChBD_CHIT1_ is flanked by two additional aromatic residues (Phe419 and Trp465), on loops L1 and L6, respectively (Fig 3A). Trp465 is a highly conserved aromatic residue across invertebrates and vertebrates in the CBM14 family (Fig 3C, S5 and S6 Figs), and adopts a surface-solvent exposed conformation closely similar to the conformation detected in the binding interface of many known structures of CBMs [55, 57, 60–62] (Fig 3A). Even though the CBM14 family is classified as a type C CBM, with lectin-like properties that optimally bind to mono-, di-, or tri-saccharides [63–64], ChBD_CHIT1_ displays a high affinity to crystalline chitin as demonstrated in *in vitro* experiments [65]. This property belongs to CBM type A and is characterized by the presence of a “flat platform” which interacts with the planar polycrystalline chitin [63]. Importantly, a construct of ChBD_CHIT1_ lacking Trp465 and Asn466 completely loses the binding activity towards chitin [66]. Taken together, we propose that Trp465 plays a key role in the binding of the ChBD_CHIT1_ to the chitinous crystalline surface probably assisted by the presence of other aromatic residues in the core of ChBD_CHIT1_ that increase the overall hydrophobic character of this domain thus increasing the affinity for the crystalline chitin.

To gain more insight into ChBD_CHIT1_ mode of action, we have also investigated its electrostatic surface properties. Interestingly, the ChBD_CHIT1_ domain reveals two different charged faces. One face is mostly neutral with a negatively charged spot spanning two residues (Ser439, Asp425), while the second face is highly positively charged mainly due to the presence of four basic residues (Arg444, Arg434, Lys423 and Lys461) (Fig 3D). These residues could potentially form hydrogen bonds with the hydroxyl groups and N-acetyl group of the bound chitin chain, strengthening the interaction. This issue will be subject of further studies.

## 4. Conclusion

In this study, we report an original crystallization approach for obtaining the full length structure of the human chitinase, CHIT1. The lack of electron density corresponding to the hinge region linking the catalytic domain to the ChBD prompts us to suggest a high flexibility of this region resulting in a random positioning of the entire ChBD. The structure of ChBD_CHIT1_ reveals a distorted β-sandwich fold which appears to be conserved within the CBM14 family across invertebrates and humans. Indeed, our data draw attention to the structural similarity between the human ChBD_CHIT1_, tachycitin and house dust mite allergen proteins. In these ChDB modules, the highly conserved cysteine residues have an essential role in maintaining the functional conformation of the domain by forming disulfide bridges. The investigation of the aromatic ring pattern of ChBD_CHIT1_ reveals that the binding interface contains a conserved aromatic residue (Trp465) adopting a surface-exposed conformation, that might enable efficient binding to sugar moieties. Furthermore, the ChBD_CHIT1_ presents a positively charged surface which could be involved in electrostatic interaction. Finally, we believe that our developed crystallization methodology could be used for co-crystallization or soaking experiments with different ChBD substrates or for solving the still elusive 3D structure of AMCase-FL and other CAZyme-CBM proteins. In conclusion, our results have revealed novel structural aspects of human ChBDs which give new insights into their characteristics.

## Supporting Information

S1 Fig CHIT1-FL sample analysis. (A) 12% SDS of the protein sample after migraion and stained by Coomassie Brillant Blue. Lane **a**–contains molecular weight standards, lane **b**–purified CHIT1-FL sample and lane **C**—dissolved CHIT1-FL crystals from condition F6. (B) A chromatogram shows the elution peak during purification of the CHIT1-FL by size-exclusion chromatography. (D) Negative-ion mode ESI-MS spectrum of the native CHIT1-FL. The negative ion peaks with m/z ratios of 50800 Da correlate with the monomer form of CHIT1-FL which has a molecular weight of 51051.3 Da.
(TIF)

S2 Fig Model of CHIT1-FL and electron density maps (1σ cutoff) in the asymmetric unit (2F_o_-F_c_ map—grey, F_o_-F_c_ map—green). (TIF)

S3 Fig Data after submitting the structure coordinates in PDBe server PISA (Protein Interfaces, Surfaces and Assemblies). (TIF)

S4 Fig Representation of the thermal parameter distribution shown as B-factor `putty' as implemented in PyMOL (http://www.pymol.org). A) CHIT1-FL with a zoom on the ChBD_CHIT1_. B) ChiB from *Serratia marcescens* with a zoom on the hinge and the ChBD_ChiB_. The Calpha-atom B-factors are depicted on the structure in dark blue (lowest B-factor) through to red (highest B-factor), with the radius of the ribbon increasing from low to high B-factor.
(TIF)

S5 Fig Sequence alignment and conservation of ChBD homologues of ChBD_CHIT1_. Color-codes depend on the residue conservation degree (conserved, magenta to variable, cyan).
(TIF)

S6 Fig Sequence alignment and conservation of ChBD homologues of ChBD_CHIT1_. Color-codes depend on the residue conservation degree (conserved, magenta to variable, cyan).
(TIF)
