# Supplementary figures and images for: X-Ray Crystal Structure of the Full Length Human Chitotriosidase (CHIT1) Reveals Features of Its Chitin Binding Domain
Source: PLoS One. 2016 Apr 25;11(4):e0154190. doi: 10.1371/journal.pone.0154190 (PMC4844120; doi:10.1371/journal.pone.0154190)

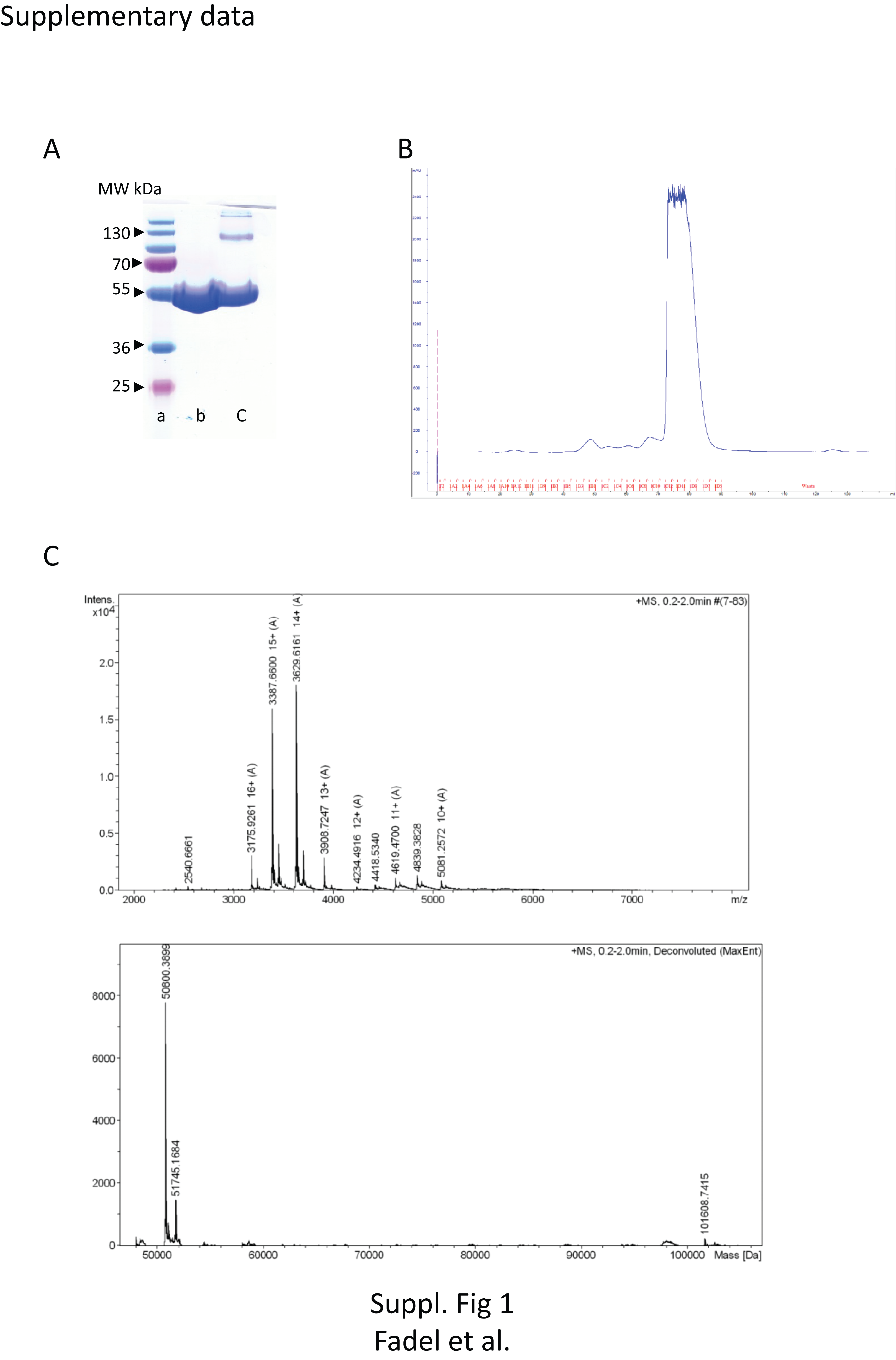

Supplement: S1 Fig — (A) 12% SDS of the protein sample after migraion and stained by Coomassie Brillant Blue. Lane a–contains molecular weight standards, lane b–purified CHIT1-FL sample and lane C—dissolved CHIT1-FL crystals from condition F6. (B) A chromatogram shows the elution peak during purification of the CHIT1-FL by size-exclusion chromatography. (D) Negative-ion mode ESI-MS spectrum of the native CHIT1-FL. The negative ion peaks with m/z ratios of 50800 Da correlate with the monomer form of CHIT1-FL which has a molecular weight of 51051.3 Da. (TIF) [file pone.0154190.s001.tif]

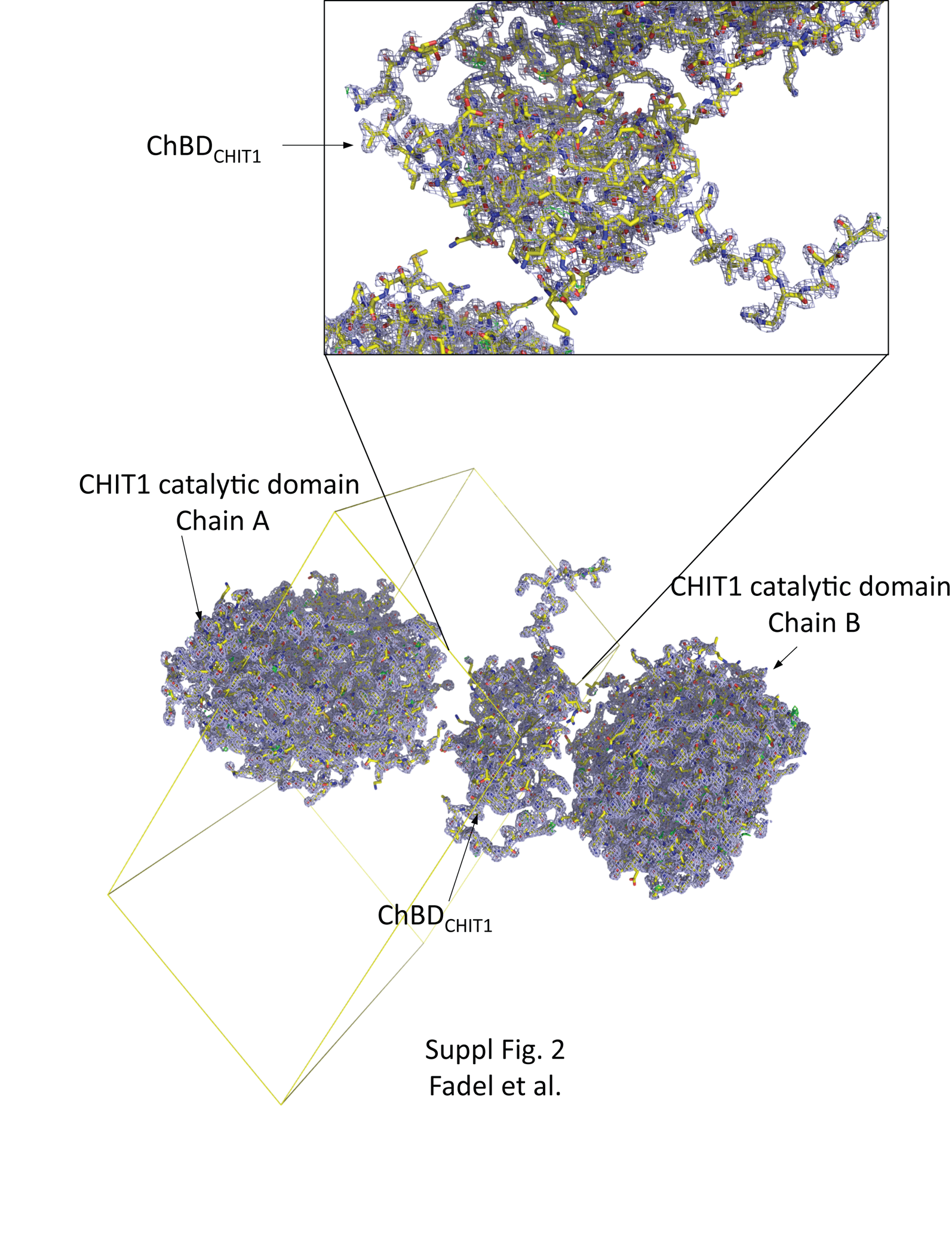

Supplement: S2 Fig — (TIF) [file pone.0154190.s002.tif]

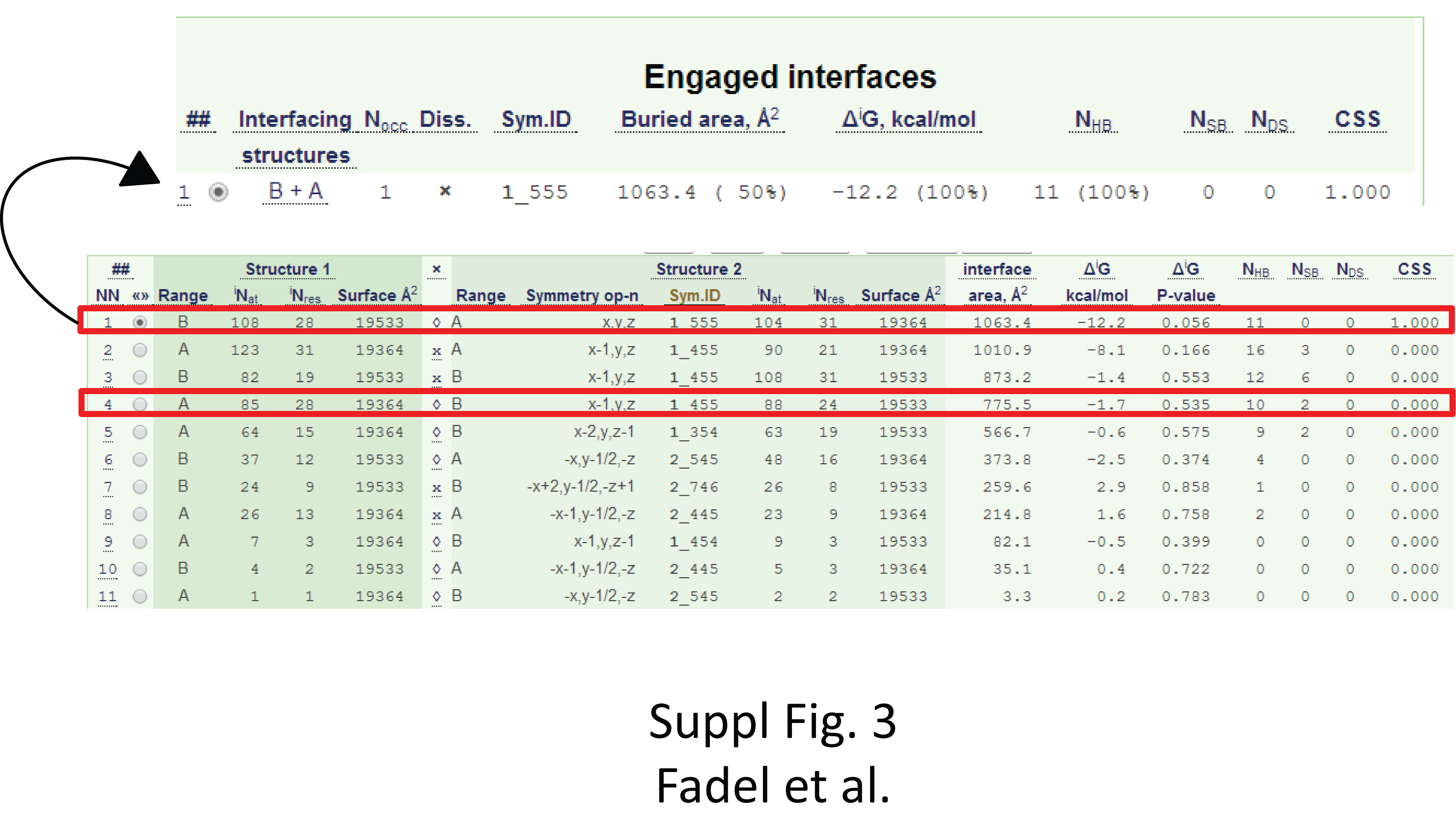

Supplement: S3 Fig — (TIF) [file pone.0154190.s003.tif]

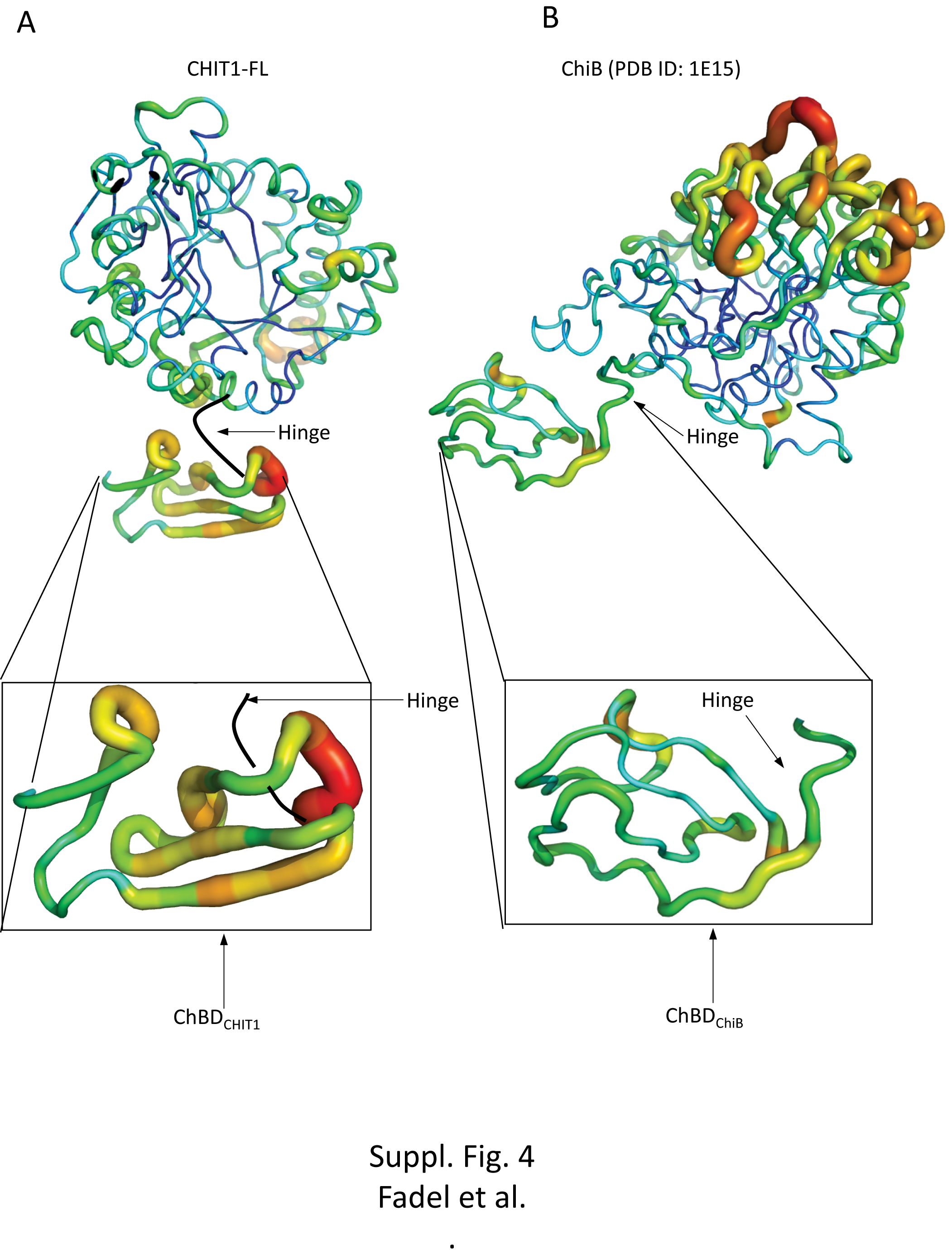

Supplement: S4 Fig — A) CHIT1-FL with a zoom on the ChBDCHIT1. B) ChiB from Serratia marcescens with a zoom on the hinge and the ChBDChiB. The Calpha-atom B-factors are depicted on the structure in dark blue (lowest B-factor) through to red (highest B-factor), with the radius of the ribbon increasing from low to high B-factor. (TIF) [file pone.0154190.s004.tif]

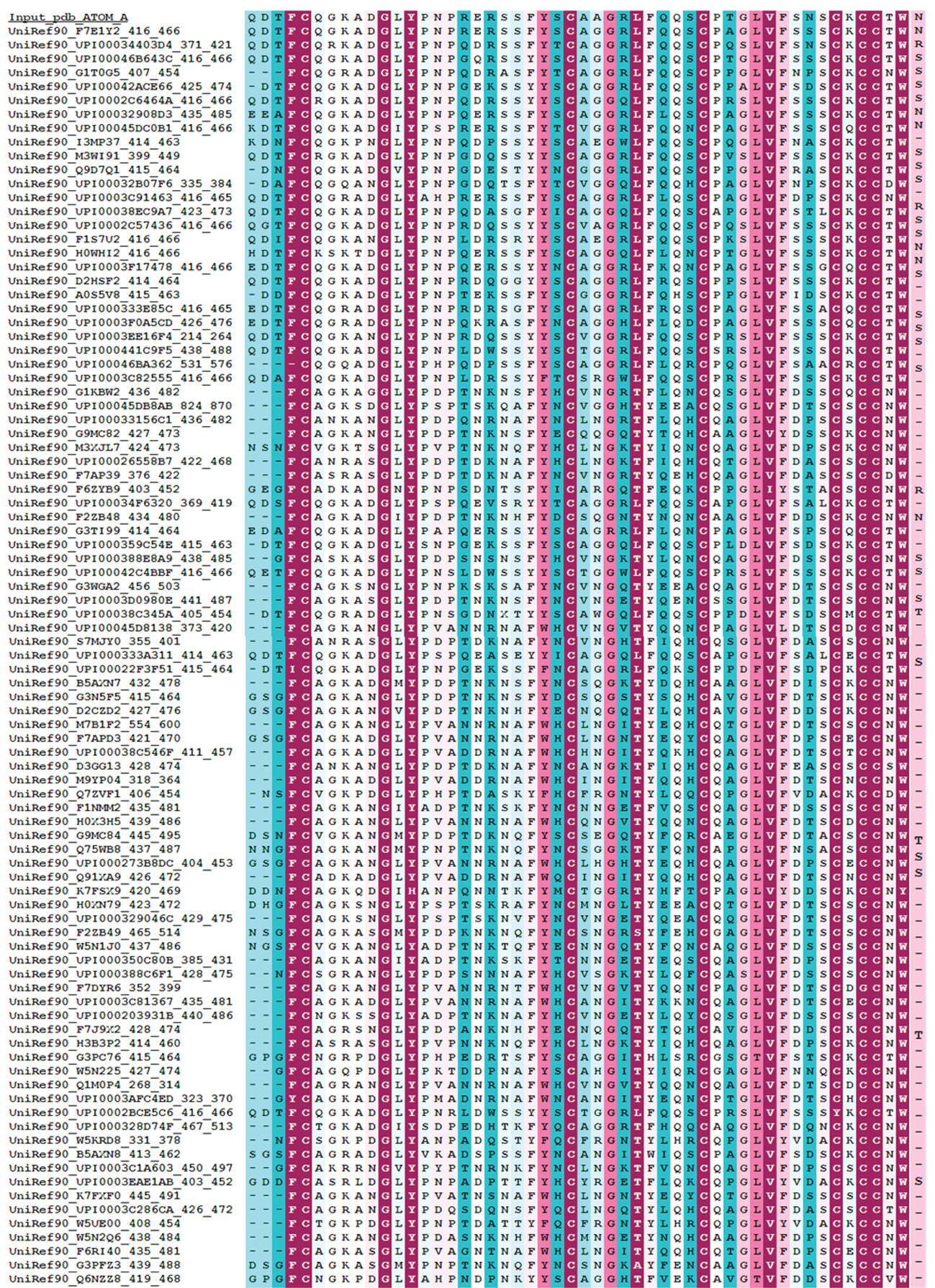

Supplement: S5 Fig — Color-codes depend on the residue conservation degree (conserved, magenta to variable, cyan). (TIF) [file pone.0154190.s005.tif]

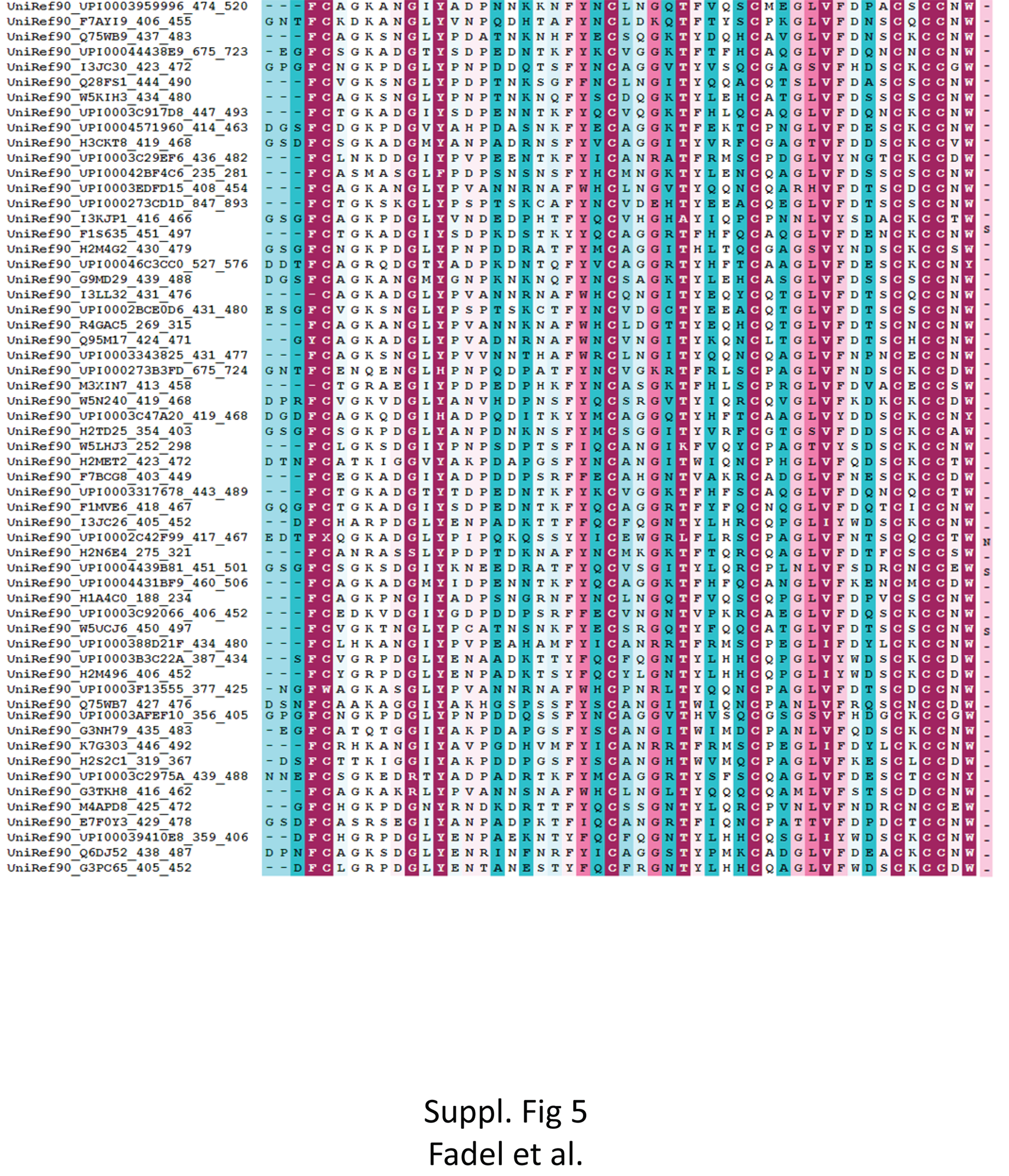

Supplement: S6 Fig — Color-codes depend on the residue conservation degree (conserved, magenta to variable, cyan). (TIF) [file pone.0154190.s006.tif]
